# How Past and Present Influence the Foraging of Clonal Plants?

**DOI:** 10.1371/journal.pone.0038288

**Published:** 2012-06-01

**Authors:** Philipe Louâpre, Anne-Kristel Bittebière, Bernard Clément, Jean-Sébastien Pierre, Cendrine Mony

**Affiliations:** UMR CNRS 6553 ECOBIO/UMS 3343 OSUR, University of Rennes I, Rennes, France; University of Tartu, Estonia

## Abstract

Clonal plants spreading horizontally and forming a network structure of ramets exhibit complex growth patterns to maximize resource uptake from the environment. They respond to spatial heterogeneity by changing their internode length or branching frequency. Ramets definitively root in the soil but stay interconnected for a varying period of time thus allowing an exchange of spatial and temporal information. We quantified the foraging response of clonal plants depending on the local soil quality sampled by the rooting ramet (*i.e.* the present information) and the resource variability sampled by the older ramets (*i.e.* the past information). We demonstrated that two related species, *Potentilla reptans* and *P. anserina*, responded similarly to the local quality of their environment by decreasing their internode length in response to nutrient-rich soil. Only *P. reptans* responded to resource variability by decreasing its internode length. In both species, the experience acquired by older ramets influenced the plastic response of new rooted ramets: the internode length between ramets depended not only on the soil quality locally sampled but also on the soil quality previously sampled by older ramets. We quantified the effect of the information perceived at different time and space on the foraging behavior of clonal plants by showing a non-linear response of the ramet rooting in the soil of a given quality. These data suggest that the decision to grow a stolon or to root a ramet at a given distance from the older ramet results from the integration of the past and present information about the richness and the variability of the environment.

## Introduction

Most mobile organisms face spatial and temporal heterogeneity during their lifetime. They develop adaptive plasticity in order to forage efficiently on these patchy environments. Plants, especially clonal species, cope with such heterogeneity of nutrients, light, and water [1]–[3]. Most of them develop as a network structure of ramets connected by stem-derived organs (stolons or rhizoms) which favors their mobility and exchange of information between ramets during plant growth [4]. Foraging may be achieved through (i) space exploration and exploitation by exhibiting particular architectural traits such as branching frequency, internode length or branching angle and (ii) resource uptake optimization through ramet specialization. Ramets may indeed change their morphological traits or mass allocation to the organs responsible for resource harvesting in order to uptake the most abundant resource of the patch [5]–[8]. Both processes may be adaptive since these clonal traits have an effect on the plant performances by modifying their biomass or abundance when they face heterogeneity [9]–[11]. These foraging strategies of clonal plants have been compared to foraging behavior of animals since the 1980s [8], [12]. They continue to inspire research on plant behavior because of their diversity, the environmental constraints they face and their clonal pattern [13]–[15].

Like other organisms, clonal plants are not omniscient about their environment. Clonal plants are then likely to use environmental stimuli in order to forage adaptively. Different types of stimuli may inform plants about the quality of their environment. For example, the quality of a given area could be estimated by (i) the amount of available resource and (ii) the spatial and temporal variability of the resource distribution. (i) The amount of available resource may be used by clonal plants as an indicator of local environment quality. Internode length between two ramets should decrease in a rich environment, leading to daughter ramets aggregation in favorable area. Alternatively, internode length should increase to avoid an unfavorable area of low resource availability. These strategies were shown in many clonal plants species [8] in response to light [16]–[18] or nutrients [19]. (ii) The spatial and temporal variability of resource is also important to consider because extending connections in a highly variable and unpredictable environment can be risky. For example, the spatial variability of nutrients informs about the expected levels of soil quality at a given distance from the ramet, thus making the environment more or less predictable [20]. Intuitively, internode length should be shorter in a highly variable environment because it is not necessary to produce a long internode to escape an unfavorable area. Under more homogeneous conditions, the environment is more predictable even at longer distances and the clone behavior should be risk prone by increasing internode lengths. Facing the spatial and temporal resource variability, clonal plants respond by inducing directional growth and resource translocation between ramets growing within sites of different qualities or by capturing unpredictable resources at different time [5], [21]–[23].

Annual plants non-additively integrate information about their environment (*e.g.* local soil quality and the presence of neighbors) to adjust their rooting [15]. Clonal plants are also likely to share information about environment quality and variability [23]–[25], which may help them to respond adaptively [26]. The interconnected ramets share water and nutrients. This physiological integration among ramets characterizes clonal plants by importing resources toward resource-deficient ramets and allowing them to perceive and respond to information from different points of the environment. In this sense, the local soil quality refers to *present information* sampled by a rooted ramet while the resource variability refers to *past information* sampled by older ramets. Sharing information through the network structure is thus likely to be an adaptive response to spatial and temporal heterogeneity because past and present information is likely to be shared among ramets in order to adjust the growth pattern of the clonal plant [27]. Little is known however about how clonal plants sample and synthesize information sampled at different space and time by the network structure.

In this study, we distinguished the effect of information about soil quality and variability on the foraging behavior of two clonal plant species growing under artificial environments. We hypothesized the following: (i) clonal plants respond to soil quality by decreasing internode length in favorable area and increasing it in unfavorable ones; (ii) this foraging response depends on the resource variability, expecting a decrease in internode length with an increase in environment variability; (iii) the foraging response to present information depends on the experience acquired by older ramets of the clone because past information is shared among ramets. We assessed the effects of two types of information (the soil quality and variability) on the foraging behavior of clonal plants by measuring internode lengths of a primary linear stolon between ramets growing in separate pots. We subsequently determined the behavioral response of the youngest ramets relatively to the experience of older sampling ramets.

## Materials and Methods

### The biological material

Two closely related stoloniferous species, *Potentilla reptans* L. and *P. anserina* L. (*Argentina anserina* L. Rvdb) (Rosaceae) were chosen. A close phylogenetic relationship between the species should prevent a broad range of treatment responses. Adult rosettes of both species form long, sympodial stolons with rooted ramets at each node [24], [28], [29]. Rooting occurs if the lower part of the stolon node contacts moist soil (Louâpre and Bittebiere, personal observations). Internodes are usually 10 to 20 cm long, depending on environmental conditions [29], [30]. In the absence of physical disturbance, ramets remain connected throughout one growing season [31]. The two species are commonly distributed in disturbed habitats, including grazed grasslands, road margins, and lake and river shorelines [24], [28]. *Potentilla anserina* also occurs on seashores and in brackish marshes [28], [32].

### Pre-treatment conditions

In winter 2009, a total of 12 clonal fragments of *P. anserina* and *P. reptans* were randomly collected in Western France from two common sites and one additional site per species (*i.e.* four sampling sites in total but only three sites per species). The sites were chosen to represent typical habitats for the species, and included mown and grazed meadows, and wet oligotrophic or eutrophic meadows. The clonal fragments were cultivated for six months under uniform outdoor conditions in trays filled with a substrate of medium quality (50% sand and 50% compost, see below) in the experimental garden at the University of Rennes 1 (France). New ramets were watered every two days to prevent water stress. On July 1^st^ 2010, we removed four ramets with one internode connection from each of the 12 pre-cultivated clonal fragments from the two species (12 replicates per treatment for a total of 96 ramets). Removed ramets were of similar size and age.

### The experiment

**Figure 1:**
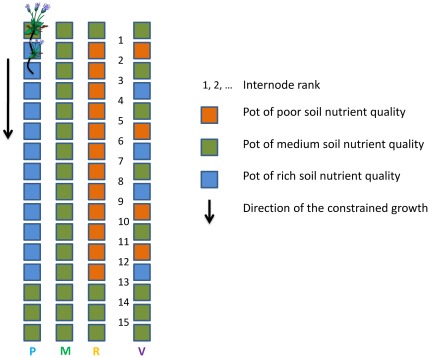
The experimental design. Clonal growth was oriented in the direction indicated by the arrow with a single ramet per pot. The letters designate the different treatments tested: P = poor, M = medium, R = rich, and V = variable (see Materials and Methods section for details).

The experiment was conducted in the experimental garden at the University of Rennes 1 (France) from the beginning of July to the beginning of October 2010. Each of the four ramets was randomly assigned to one replicate of four treatments. Each ramet was cultivated in square plastic pots (8×8×7 cm^3^) arranged in a line, each pot assigned to the cultivation of one ramet (Fig. 1). The position of the pot was moved following the stolon apex and fixed only when a ramet rooted. We tested three soil quality levels using different sand and compost mixtures: Poor (P) 3∶1, Medium (M) 1∶1, Rich (R) 1∶3, and Variable (V), comprised individual pots of P, M, and R soils randomly placed in a line (the sequence of pots thus differed between each clone to avoid potential effects of a given order). The compost contained a slow diffusing fertilizer (amounts equivalent to 0.44 kg.m^−3^ N, 0.5 kg.m^−3^ P, 0.56 kg.m^−3^ K), which ensured stable soil quality throughout the experiment. The P-, M- and R- treatments were characterized by a null variance in soil quality, whereas the V-treatment had an average quality equal to the M-treatment with a non-null variance (Fig. 1). We transplanted the initial ramet in the first pot of each line with a medium quality soil to limit transplantation stress. Soil quality in the second to 13^th^ pots corresponded to the tested treatment, and the last three pots of the line (14–16) were filled with a medium quality soil to have comparable soil nutrient conditions among treatments at the end of clonal growth (Fig. 1). Ramets were watered daily, and weeds were removed manually. During clonal growth, secondary stolons (branches developing from the primary stolon) and flowers were counted and excised to avoid diverting resources from the growth of the primary stolon. We focused on internode length independently of biomass allocation as a measure of the foraging behavior. We harvested each individual (a sequence of 16 ramets, their roots and connections) as soon as the 16^th^ ramet rooted in the last pot of the culture line, and measured each internode length. Each internode is identified by the rank of the ramet initiating it (Fig. 1).

### Statistical analysis

We analyzed internode lengths from number two to 13 using Generalized Additive Models [33]. GAM is a further generalization of the Generalized Linear Model. This method allows coping with nonlinear effects of covariates, providing they act independently of each other and of the linear terms. The principle is to fit the residuals of the linear part by smooth functions of the covariates suspected of having a non linear effect. These functions are non-parametric in the sense that no explicit algebraic function is used. Instead, a smoother is used. Generally the smoother is a basis of cubic spline functions, but other splines or other smoothing functions may be used. The method is powerful when the purpose of the analysis is to prove the effect of a covariate, even in a non linear way, and when the user is not interested in giving a precise parametric form to the non linear terms. Splines and other smoothers introduce as many parameters in the model as the number of “knots” used for smoothing. The equivalent degrees of freedom so introduced are not integers and must be computed in the fitting process. The parameters are *β* terms of regression. They have no individual signification. Therefore, they are not tested individually against zero, and are not exhibited in the results of most softwares. GAMs are also a powerful method for removing a trend in the data and therefore cut off possible autocorrelation. It was used here to suppress the systematic effect of the order of the ramets along the spacer. Autocorrelation of residuals was avoided by including the previous internode length as covariate. The absence of significant autocorrelation was verified using the *acf* function (Stats package RTM 2.13.1). In our study, GAM facilitated internode length prediction by estimating unspecific functions of predictor variables, including treatment or prior plant response. We studied the effects of (i) soil quality and (ii) variability on the response exhibited by the two species, regardless of the response shape. We included clonal fragment origin as categorical variable in the two models.

Generalized Linear Models (Poisson distribution with a log link function) were used to analyze two other responses; flower and secondary connection numbers excised during the experiment.

The last three internode lengths (number 13 to 15) were independently analyzed. These data were collected on ramets growing in M soil, the mother ramets experiencing different soil conditions (P-, M-, R- V- treatments). We used Generalized Estimated Equations, including the pot series as a clumped factor, and Tukey's HSD post hoc test (Tukey's Honestly Significant Difference). Repeated measures analyses followed Zeger and Liang [34].

All analyses were carried out using R 2.11 software (GAM package RTM 1.06.2 and GEE Package Geepack RTM 2.13.1).

### Ethics statement

No specific permits were required for the described experimental field studies: a) no specific permissions were required for these locations/activities; b) locations are not privately-owned or protected; c) the field studies did not involve endangered or protected species.

## Results

### Effect of soil quality on ramet's foraging behavior

**Figure 2:**
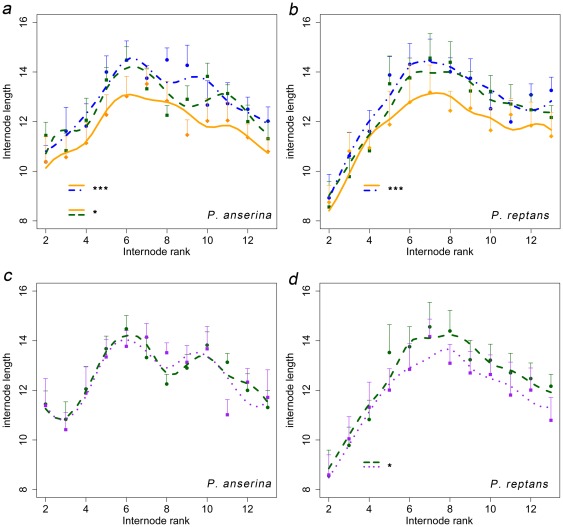
The effect of soil quality and variability on ramet's foraging behavior. Mean internode length (± Standard Error) of *P. anserina* (*a*, *c*) and *P. reptans* (*b*, *d*) from rank 2 to 13 depending on the soil quality and the resource variability (dot dashed blue = Poor, solid green = Medium, dotted orange = Rich, dotted purple = Medium Variable). Significant differences: * (*P*-value<0.05) and *** (*P*-value<0.001).

Mean internode length increased from rank 2 to 6 or 7 in both *Potentilla* species, and subsequently stabilized or decreased regardless of the treatment (internodes from rank 6 or 7 to 13) (Fig. 2a and 2b). Soil quality showed a significant effect on internode length in *P. anserina* (F_2, 347.5_ = 4.3, *P*<0.05), and *P. reptans* (F_2, 374.2_ = 3.5, *P*<0.05) (the estimated coefficient of GAM's are reported in table 1). In *P. anserina* and in *P. reptans*, shorter internode lengths were observed in the R-treatment in comparison with the P-treatment (*P*<0.001 and *P*<0.01 respectively) (Fig. 2a and 2b). In *P. anserina*, shorter internode lengths were observed in the R-treatment in comparison with the M-treatment (*P*<0.05) (Fig. 2a). The clone origin and previous internode lengths also influenced the following internode lengths in *P. anserina* (F_2, 347.5_ = 12.1, *P*<0.001, and F_2, 374.2_ = 27.7, *P*<0.001, respectively), and *P. reptans* (F_1, 347.5_ = 82.5, *P*<0.001, and F_1, 374.2_ = 61, *P*<0.001, respectively) (Table 1).

**Table 1:** Generalized additive equations showing the effects of soil quality and clone origin on internode length of *P. anserina* and *P. reptans* (from ramets 2 to 13).

Species	Factor/Covariable	modality	β	SE	t	*P*-value
*Potentilla anserina*	Treatment	Medium	0.000	0.000		
		Poor	0.132	0.246	0.537	0.591
		Rich	−0.529	0.244	−2.166	0.031
	Clone's origin	Site A	0.000	0.000		
		Site C	−0.831	0.243	−3.418	<0.001
		Site B	−1.240	0.259	−4.784	<0.001
	Previous internode length		0.426	0.047	9.083	<0.001
	Smoothing parameter: Internode Rank (F_7.9, 346.1_ = 4.8, *P*<0.001)					
*Potentilla reptans*	Treatment	Medium	0.000	0.000		
		Poor	0.262	0.251	1.043	0.297
		Rich	−0.431	0.253	−1.702	0.047
	Clone's origin	Site D	0.000	0.000		
		Site A	1.684	0.295	5.704	<0.001
		Site B	−0.781	0.256	−3.043	<0.01
	Previous internode length		0.379	0.048	7.806	<0.001
	Smoothing parameter: Internode Rank (F_4.6, 374.4_ = 6.8, *P*<0.001)					

β: estimated regression coefficients; SE: β standard error; *P*-value: β significance. *: For the smoothing parameter, degrees of freedom are estimated.

The flower and secondary connection number excised during the experiment were not influenced by different soil fertilities in *P. reptans* (χ^2^ = 10.7, *df* = 12, P = 0.55, and χ^2^ = 48.1, *df* = 50, *P* = 0.55) and *P. anserina* (χ^2^ = 24.9, *df* = 24, *P* = 0.41, and χ^2^ = 34.8, *df* = 38, *P* = 0.61, respectively).

### Effect of resource variability on ramet's foraging behavior

Resource variability modified ramet response in *P. reptans*, but not in *P. anserina* (F_1, 267.7_ = 4.1, *P*<0.05, F_1, 178.9_ = 0.2, *P*>0.05, respectively). In *P. reptans*, internode length decreased under V-treatment conditions relatively to the M-treatment (Table 2). This decrease appears graphically only after the 5^th^ internode (Fig. 2d). Clone origin and previous internode length exhibited significant effects on the following internode lengths in *P. reptans* (F_2, 178.9_ = 4.1, *P*<0.05; F_1, 178.9_ = 24.5, *P*<0.001, respectively), and *P. anserina* (F_2, 267.7_ = 19.5, *P*<0.001; F_1, 267.7_ = 48.7, *P*<0.001, respectively) grown under V- treatment soil conditions.

**Table 2:** Generalized additive equations showing the effects of resource variability, and clone origin on internode length of *P. anserina* and *P. reptans* (from ramets 2 to 13).

Species	Factor/Covariable	modality	β	SE	t	*P*-value
*Potentilla anserina*	Treatment	Medium	0.000	0.000		
		Variable	−0.115	0.252	−0.456	0.649
	Clone's origin	Site A	0.000	0.000		
		Site C	−0.038	0.305	−1.266	0.207
		Site B	−0.903	0.315	−2.865	<0.01
	Previous internode length		0.340	0.068	4.946	<0.001
	Smoothing parameter: Internode Rank (F_8.8, 179.2_ = 4.6, *P*<0.001)					
*Potentilla reptans*	Treatment	Medium	0.000	0.000		
		Variable	−0.508	0.250	−2.035	<0.05
	Clone's origin	Site D	0.000	0.000		
		Site A	1.632	0.354	4.610	<0.001
		Site B	−0.887	0.314	−2.825	<0.01
	Previous internode length		0.406	0.593	12.6.984	<0.001
	Smoothing parameter: Internode Rank (F_4.1, 267.9_ = 7.2, *P*<0.001)					

β: estimated regression coefficients; SE: β standard error; *P*-value: β significance. *: For the smoothing parameter, degrees of freedom are estimated.

Flowers and secondary connections removed during the experiment did not significantly differ between the M- and V-treatments in *P. reptans* (χ^2^ = 2.70, *df* = 5, *P*>0.05, and χ^2^ = 23, *df* = 22, *P*>0.05, respectively), and *P. anserina* (χ^2^ = 7.8, *df* = 8, *P*>0.05, and χ^2^ = 11.9, *df* = 13, *P*>0.05, respectively).

### Older ramets effect on younger ramet foraging behavior

**Figure 3:**
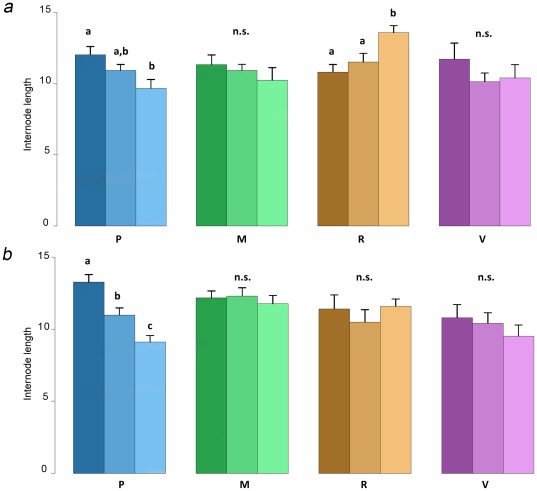
Older ramets effect on younger ramet foraging behavior. Mean internode length (± Standard Error) of *P. anserina* (*a*) and *P. reptans* (*b*) from rank 13 to 15 (from dark to light colors) in the four treatments (blue = Poor, green = Medium, orange = Rich, purple = Variable).

The effect of environmental information sampled by older ramets on the foraging behavior of younger ones in *P. anserina* and *P. reptans* was tested by comparing internode lengths from rank 13 to 16 (Fig. 3). These last three internode lengths were dependent on the treatment in *P. anserina* and *P. reptans* (GEE, interaction between internode rank and treatment: χ^2^ = 44.2, *df* = 6, *P*<0.001; χ^2^ = 28.1, *df* = 6, *P*<0.001, respectively). The last three internode lengths in *P. anserina* did not vary after growth in the M- or V-treatments, whereas results showed respectively decreased and increased internode lengths following P- and R-treatment conditions (Fig. 3a). The last three internode lengths in *P. reptans* remained constant in the M-, R-, and V-treatments, and decreased significantly in the P-treatment (Fig. 3b).

## Discussion

Under our experimental conditions, internode lengths of a primary stolon characterized part of the foraging response and should be completed by the analysis of ramet specialization. We demonstrated that both the soil quality and the resource variability influence internode lengths and that past and present information were determining for the foraging response. However, internode plasticity was limited, regardless of the nutrient resource, and influenced by previous internode length. Internode growth was therefore an interplay between foraging behavior, structural constraints, and resource availability [35]. Clone origins also determined plant response by influencing internode length in the two species.

### Local soil quality and variability as determinants of present and past information for clonal plants

The two species responded to soil quality similarly; internodes were shorter when the soil quality was high. This adaptive behavior facilitates ramet aggregation to consolidate occupation of favorable patches, and maximizes resource acquisition, as reported in *Glechoma hederacea* [19], [36]. Our results thus confirm that information about soil quality is perceived by ramets (*i.e.* the present information) which then adjust their behavior accordingly.

Resource variability was only detected as environmental information in *P. reptans*, leading to a decrease of internode length in the variable treatment. To built its response to the resource variability, *P. reptans* disposed of a local signal regarding soil quality (the present information sampled during the ramet rooting), but also of information acquired by older ramets (the past information). *Potentilla reptans* thus synthesizes present and past information in order to adjust its growth pattern to the nutrient distribution. We noted that the differences in the internode lengths between clones exploring the homogeneous and the variable treatments of the same overall medium quality appears graphically only after the 5^th^ internode. This suggests that *P. reptans* may need to have enough benchmarks to perceive and respond to the resource variability. Nevertheless, no differences were found between these two treatments when *P. reptans* reaches the last three pots of medium soil quality. As we hypothesized, an increase of internode length should be observed when clonal plants sample a more homogeneous soil because it is not necessary to produce a long connection to reach a different but more profitable area. In our experiment, we did not observe an elongation of the internode length, just a break in their decrease suggesting that *P. reptans* perceived the decrease in resource variability. Additional sampling points may tend to the expected clone response of internode lengthening.

*P. anserina* and *P. reptans* differed in their response to soil variability. A difference may exist in their integration distances such as those demonstrated in *Potentilla* species and within *Fragaria chiloensis* (Rosaceae) genotypes [37], [38]. *Potentilla reptans* may display a shorter physiological integration distance than *P. anserina*, in which the resource variability would be integrated along the entire stolon. *Potentilla anserina* consequently displayed a similar response in the Medium and Variable treatment.

### Past and present information lead to the relative perception of soil quality

Considering the homogeneous environments, internode length was dependent on the soil quality locally sampled by ramet but also on the soil quality previously assessed by older ramets. Internode length decreased (in both species) or increased (only in *P. anserina*) in medium quality soil after having experienced soils of poor or rich qualities respectively. The absence of response in *P. reptans* from rich to medium soil conditions may be due to lower soil nutrient requirements for growth compared to *P. anserina*. Indeed, preferendum of environmental quality in *P. reptans* is slightly lower than in *P. anserina* [39].

The experience dependent behavior of clonal plants may be adaptive when the soil quality of patches is not well known. When the resource distribution varies in space and time, a given amount of nutrient has a relative profitability regarding the global quality of the environment. In our experiment, the medium soil is of higher profitability than the poor soil but is of lower profitability than the rich soil. The effect of conditions under which older ramets rooted on subsequent growth has already been investigated in different situations [40]–[42]. Our results quantify this past information effect by showing a non-linear response of ramets. This suggests that the clone decision to elongate its connections or to establish a ramet at a given distance from the older ramet results from the integration of the past and present information about environmental richness.

### Past information: the dual effect of the clone experience and origin

The response of a clonal plant depends not only on the information sampled during the plant growth (present information and past information in this study) but also on the clonal origin (past information acquired before starting the experiment, what behavioral ecologists call a “genetic knowledge of the environment” in animals [43]). We thus make a distinction between the information acquired by ramets during the experiment and the genetic information characterizing the clonal fragment we used in this study. We may have selected several genotypes determining different foraging behaviors; pre-treatment conditions could not erase such genetic effect. Genetic variations may explain differences in the observed foraging behaviors by a strong interaction with the environment where the clonal plants evolved [44]. Life history traits of clonal plants are known to vary among populations facing different ramet densities [45], plant communities [46], resource richness and heterogeneity [47]. In our case, different environments are likely to have selected different trait values in response to heterogeneity in the resource distribution or to global soil quality. The three field sites where the clonal fragments of *P. anserina* and *P. reptans* were respectively collected did not differ in their mean resource availability (as defined by the Ellenberg indicator system, the term N-values of productivity are respectively of 6.2; 6.3; 6.1 and 5.9; 5.0; 5.2) [39], [48]. They may differ in their spatial and temporal variability of nutrients at the clonal fragment scale. However, data characterizing the resource variability in each sampling site are lacking. We hypothesize that clonal fragments collected in environments of high spatial and temporal variability may exhibit a stronger tendency to reduce the internode length than the ones collected in more homogeneous conditions, regardless of the nutrients availability. We cannot assume that the clonal fragments collected have expressed adaptive phenotypic plasticity under selection pressure. Because our experiment was designed to study the influence of information actively used by clonal plants, future studies involving the effect of the clonal origin are needed to quantify the part of the genetic information and the sampling information on the foraging behavior of clonal plants.

### Processing past and present information through clonal integration

One limitation of information processing in clonal plants is the lack of a nervous system [49]. However, the network structure of clonal plants composed of ramets and spacers may exempt them from requiring a proper complex nervous system. Indeed, any network of interconnected modules (neurons, ramets) seems capable of such processes [26], [50]. Because ramets stay interconnected in space and time, the information about richness at a given point of the clonal network may be shared with other ramets, thus resulting in a potentially adaptive response through the whole plant experience. An interconnected network of ramets is also the necessary condition to perceive any spatial variability for an organism that does not move itself. In that sense, minimal cognition consisting of perceiving and responding to the spatiotemporal environmental features [51] may be applied to clonal plants.

Information sharing through clonal integration is known to occur in clonal plants [23], [25] though little is known on the way this transfer of information occurs. Information transduction may be performed through plant hormones (auxin or abscisic acid), or resource molecules (sugar or ionic nutrients) [52], [53]. We demonstrated a non-linear response of clones to soil quality; information should thus be perceived and integrated at each node of the network. The level of information exchanging depends on the integration distance between ramets. This variable integration distance is a memory-like process of spatial and temporal information making useful the clonal network structure: A longer integration distance may mimic a long-term memory of the resource variability while a shorter integration distance may mimic a short-term memory. Future studies are needed to investigate the relationship between variable resource distribution and integration distance, expecting that the more variable the environment is, the longer the integration distance should be.

McNickle *et al.* [13] argued for a new conceptual foundation of optimality in plant foraging behavior. We demonstrated here that complex integration of different information should inspire proximal mechanism studies of information processed by clonal plants in the broader field of behavioral ecology. The effect of plant experience on ramet behavior takes the comparison with higher cognitive center such as nervous system a step further. This suggests for example similarities between animal memory and clonal integration, opening the field of foraging behavior in clonal plants to embark on several more avenues of investigation.
